# Production of Succinic Acid by Metabolically Engineered *Actinobacillus succinogenes* from Lignocellulosic Hydrolysate Derived from Barley Straw

**DOI:** 10.4014/jmb.2410.10053

**Published:** 2024-11-25

**Authors:** Bo-Kyung Kim, Min-Seo Park, Minseok Cha, Young-Lok Cha, Soo-Jung Kim

**Affiliations:** 1 Department of Integrative Food, Bioscience and Biotechnology, Chonnam National University, Gwangju 61186, Republic of Korea; 2 Research Center for Biological Cybernetics, Chonnam National University, Gwangju 61186, Republic of Korea; 3 Bioenergy Crop Research Institute, National Institute of Crop Science, Rural Development Administration, Muan 58545, Republic of Korea

**Keywords:** Succinic acid, *Actinobacillus succinogenes*, metabolic engineering, barley straw, lignocellulosic biomass

## Abstract

Succinic acid is an industrially important component that plays a key role in food additives, dietary supplements, and precursors for biodegradable polymers. Due to environmental and economic issues, succinic acid production has become increasingly attractive. This work aimed to improve succinic acid production from lignocellulosic biomass in *Actinobacillus succinogenes* through genetic modifications and fermentation strategies. Firstly, the effects on succinic acid production by overexpressing genes encoding phosphoenol carboxylase, malate dehydrogenase, and fumarase were evaluated in batch fermentations of engineered *A. succinogenes* strains. The engineered *A. succinogenes* expressing PCK, MDH, and FUM (AS-PMF) showed a 1.3-fold increase in succinic acid production compared to the wild-type strain. Subsequently, the fed-batch fermentation with MgCO_3_ was carried out using AS-PMF, which led to producing 50 g/l of succinic acid with 0.79 g/g of yield. Finally, 22.2 g/l of succinic acid with 0.64 g/g of yield was achieved in batch fermentation from lignocellulosic hydrolysate of barley straw. These results support that sustainable succinic acid from agricultural wastes might be a promising strategy for industrial applications.

## Introduction

Microorganisms are able to produce a variety of valuable chemicals from lignocellulosic biomass, such as switchgrass, barely straw, rice straw, etc., which can be sustainable feedstocks that should be a promising sustainable method for environmental and economic purposes [1-4]. Among these chemicals, succinic acid (SA) is an important organic acid found in plants, animals, and even humans [5-7] that has special advantages and should be a key building block for producing a variety of value-added chemicals including adipic acid, 1,4-butanediol (BDO), poly-butyrate succinate (PBS), a wide range of food flavorings, pharmaceutical applications, cosmetics, and many other things [8, 9]. Specifically, the beneficial aspects of SA for pharmaceuticals and cosmetics should be that it has antimicrobial, anti-inflammatory, and antioxidant properties [10].

Currently, most of SA is predominantly produced from n-butane, which comes from crude oil-based feedstocks through oxidation to maleic anhydride and catalytic hydrogenation of maleic acid or maleic anhydride [11, 12]. Issues with the petrochemical-based production of SA should be that 1) this production method reported is not cost-effective and 2) it causes negative impacts on the environment [13]. Because of these reasons, the research field of microbial fermentations for SA production is growing [14]. Microbial metabolic engineering techniques are improving significantly to achieve better SA production. There are several reviews describing effective metabolic engineering techniques and microbial strains that can produce SA [15-17]. So far, the strains used for SA productions are *Actinobacillus succinogenes*, *Anaerobiospirillum succiniciproducens*, *Mannheimia succiniciproducens*, and *Escherichia coli*. *Corynebacterium* sp. and *Bacteroides fragilis* strains have been also suggested as SA platform strains [15, 18, 19]. One of the important strategies to get better SA production should be metabolic engineering, which is modifying the metabolic pathways and optimizing the growth conditions, etc., of microorganisms [20]. *E. coli* was genetically modified to enhance SA production through the SA pathway by introducing specific genes and removing the branch synthesis pathways [21]. *E. coli* has been broadly engineered for SA production by improved substrate transport, increased carbon flux, and removed branch pathways [22-24]. Furthermore, *Saccharomyces cerevisiae*, *Yarrowia lipolytica*, *A. succinogenes*, and *M. succiniciproducens* have also been genetically engineered for SA productions [25].

*A. succinogenes* is a facultative anaerobic, mesophilic, pleomorphic, Gram-negative rod, a member of the *Pasteurellaceae* family, capable of fermenting a broad spectrum of carbon sources [26, 27]. This bacterium that was isolated from bovine rumen is also capnophilic and osmotolerant, and it accumulates high titers of SA (>70 g/l)[27]. This can also ferment cane molasses, whey, and wheat hydrolysates as alternative carbon sources cheaper than refined glucose and sugars [28-30]. As previous studies reported, genes-related SA production in *A. succinogenes* should be modified by metabolic engineering techniques, and cell growth conditions should also be optimized to obtain higher amounts of SA production [31]. These strategies were focusing on the modification of the reductive TCA cycle, which was terminated by SA, the end product [32, 33]. In another previous study, the mutant strain with deactivating the function of pyruvate formate lyase (PFL) was constructed, and this constructed strain successfully and completely removed formic acid biosynthesis [34]. The constructed strain also provided the improved carbon flow to SA [34]. Although the study showed improved SA productivity, *A. succinogenes* is not easy to handle for gene manipulations. For this reason, it is hard to improve the SA production by *A. succinogenes* [31, 33]. Therefore, optimizing culture conditions in the fermentation process should be necessary.

Herein, engineered strains would be constructed to overexpress genes associated with the SA biosynthesis pathway in *A. succinogenes* to improve SA production. With the specifically designed/constructed strains, we would focus on 1) optimizing the fed-batch fermentation and 2) producing SA from a real-world substrate, lignocellulosic biomass like barley straw, as a sole carbon source.

## Materials and Methods

### Strains, Media, and Growth Conditions

*A. succinogenes* 130Z and engineered strains used in this study are listed in Table 1. *E. coli* DH5α was a host strain for constructing plasmids. *A. succinogenes* was grown on a Tryptic Soy Broth (TSB; Condal, Canada) medium containing 10 g/l glucose at 37°C and 180 rpm. Transformants were grown on TSB with 10 g/l of glucose (TSBG) plates containing 100 μg/ml ampicillin or 17 μg/ml chloramphenicol. Transformant *E. coli* strains were grown on a LB medium containing with 100 μg/ml ampicillin or 34 μg/ml chloramphenicol at 37°C and 250 rpm.

### Construction of Plasmids for the Gene Overexpression

All plasmids and primers used in this study are summarized in Tables 1 and S1/S2, respectively. To improve the production of SA, plasmids were constructed to overexpress genes involved in SA biosynthesis. The plasmid containing *mdh* or *pck* gene was purchased from Addgene (USA), while the plasmids with both *mdh* and *pck* were constructed in this study (Fig. S1). DNA fragments were amplified by polymerase chain reactions (PCR) with corresponding primers and assembled using the 2X Gibson Assembly Master Mix (NEB, USA). All PCR amplifications were performed using Phusion High-Fidelity DNA Polymerase (NEB).

A *fum* locus was amplified from an *A. succinogenes* genomic DNA with a set of primers, FUM_F and FUM_R, and a backbone DNA fragment including an ampicillin (Amp)-resistance gene and a promoter *pckA* was amplified from a PCK plasmid with a set of primers, pPCK_F and pPCK_R. The two amplified DNA fragments were assembled to generate a pFUM plasmid. To construct a pPMF, a pPCK-Cm was generated by replacing an Amp-resistance gene in the pPCK with a chloramphenicol (Cm)-resistance gene. The Cm-resistance gene was synthesized by COSMO Genetech (Republic of Korea). After that, a backbone was amplified from pPCK-Cm with primers of V_PMF-F and V_PMF-R. The insert genes of *mdh* and *fum* with their promoters were amplified from an *A. succinogenes* genomic DNA with sets of primers, MDH_FUM_R/ MDH_PCK_R and Prom-FUM-F/ Prom-FUM-R. These amplified DNA fragments were assembled to construct the plasmid of pPMF (Fig. S1). Gene sequences for *mdh*, *fum*, *pckA*, *Amp*, and *Cm* are in Table S3, and sequences for all relevant promoters used in this study are in Table S4.

### Transformation of *A. succinogenes*

Constructed plasmids were introduced into *A. succinogenes* through electroporation using a MicroPulser electroporator (Bio-Rad, USA). To prepare competent cells, cells were grown up to an optical density at 600 nm (OD_600_) of 0.4 with a working volume of 250 ml and harvested by centrifugation (6,000 ×*g*, 20 min) at room temperature. Then, the pellet was washed twice with pre-chilled 20% (v/v) glycerol. After the second wash, the cell pellet was resuspended in 2.5 ml of pre-chilled 20% (v/v) glycerol in a microcentrifuge tube. The competent cells were aliquoted into 100 μl and stored at −80°C until needed. Competent cells were mixed with 300-400 ng of DNA in a pre-chilled 2 mm cuvette and electroporated at 2.5 kV, 25 μF and 400 Ω. After pulsing, 750 μl of TSB supplemented with 10 g/l glucose was added to the cuvette. The electroporated cells in TSBG were incubated at 37°C for 1 h. Then, cells were plated on TSBG agar plates supplemented with appropriate antibiotics for 1-2 days.

### Fermentation

For pre-cultures, *A. succinogenes* strains were cultured aerobically or anaerobically in 125 ml serum bottles with a working volume of 25 ml TSBG and 17 μg/ml chloramphenicol or 100 μg/ml ampicillin at 37°C and 200 rpm for 12-18 h. For the main culture, the pre-cultured cells were cultivated for 6-8 h and then inoculated into a fresh TSBG medium. Cells were inoculated into a fermentation medium at an initial OD_600_ of 0.05. To make the culture bottles anaerobically, CO_2_ gas was purged for 30 min.

For batch fermentation, a fermentation medium consisted of 20.1 g/l tryptic soy broth, 13.4 g/l peptone, 6.7 g/l yeast extract, and carbon sources. The supplied carbon sources were 20 g/l of glucose, 20 g/l of xylose or a mixture of 10 g/l of glucose and 10 g/l of xylose. Batch fermentations were carried out in the medium with a working volume of 2 L in a 5 L bioreactor (Kobiotech, Republic of Korea) at 37°C. The pH was maintained at 6.8 by adding 6 N NaOH, and the agitation speed was set at 200 rpm. For anaerobic conditions, CO_2_ was supplied at the rate of 0.5 ml/min. Dissolved oxygen (DO) was monitored by an O_2_ sensor.

For fed-batch fermentations, the medium was prepared with 20.1 g/l tryptic soy broth, 13.4 g/l peptone, 6.8 g/l yeast extract, and a mixture of 20 g/l glucose and 20 g/l xylose. All conditions (pH, CO_2_ rate, stirrings, and temperature) in fed-batch fermentations were maintained at the same as those in batch fermentation. To test the effect of pH buffering agent on succinic acid production, 50 g/l MgCO_3_ was utilized for adjusting pH. The feeding solutions were composed 900 g/l xylose and 900 g/l glucose. When the concentration of residual glucose and xylose in the medium was reduced to below 7 g/l, the feeding solution was intermittently supplemented with at the concentration of 20 g/l glucose and 20 g/l xylose.

### Preparation of Lignocellulosic Hydrolysate from Barley Straw

The barley straw used in this study was collected in Yeonggwang, South Korea in 2021. After 48 h of natural drying, the barley straw was initially pulverized to a size of 5-10 cm and then further reduced to a 3 mm size. The organosolv process was conducted for 1 h using a mixed ethanol and NaOH solvent at a 1:9 ratio. The treated solids were neutralized and rinsed with running water. After 24 h of oven drying, the solids were stored in a desiccator. Following the established experimental procedure at NREL, a pretreated barley straw oligo reaction was conducted. The Cellic CTec 3 (Novozymes, Denmark) was used as the enzyme preparation, containing effective cellulases and β-glucosidase for biomass saccharification. Reactions were performed at a stirring speed of 180 rpm and a reaction temperature of 50°C in 50 ml Erlenmeyer flasks with a reaction volume of 20 ml. To maintain the pH level at 5.0, 1 M citrate buffer (pH 4.8) and 1 M sulfuric acid were used as pH adjusters. Additionally, polyethylene glycol 8000 (PEG, Sigma-Aldrich, USA) was added at a concentration of 3% of glucan as a saccharification enhancer.

### Determination of Cell Growth, Carbon Sources, and Metabolites

The growth of the cells was monitored by measuring the OD_600_ using a spectrophotometer (UV-1900i, Shimadzu, Japan). To measure the concentrations of glucose, xylose, SA, and other end-products, including lactic acid, acetic acid, and formic acid, the cultured broth was centrifugated at 6,000 rpm for 5 min to separate cells and supernatant. After centrifugation, the supernatant was collected and filtered with 0.22 μm syringe. The filtered and appropriately diluted supernatant was subjected to determine metabolites using high-performance liquid chromatography (HPLC; SCL-40, Shimadzu) equipped with a Rozex ROA-organic acid-H+ (8%) column (Phenomenex, USA) and a refraction index (RI) detector. It was eluted with the mobile phase, which was 5 mM H_2_SO_4_ at a flow rate of 0.6 ml/min, and the column was maintained at 60°C. Standards for quantification of metabolites were purchased from Sigma-Aldrich (Merck Korea, Republic of Korea).

## Results and Discussion

### Overexpression of SA Biosynthetic Genes in *A. succinogenes*

**Fig. 1:**
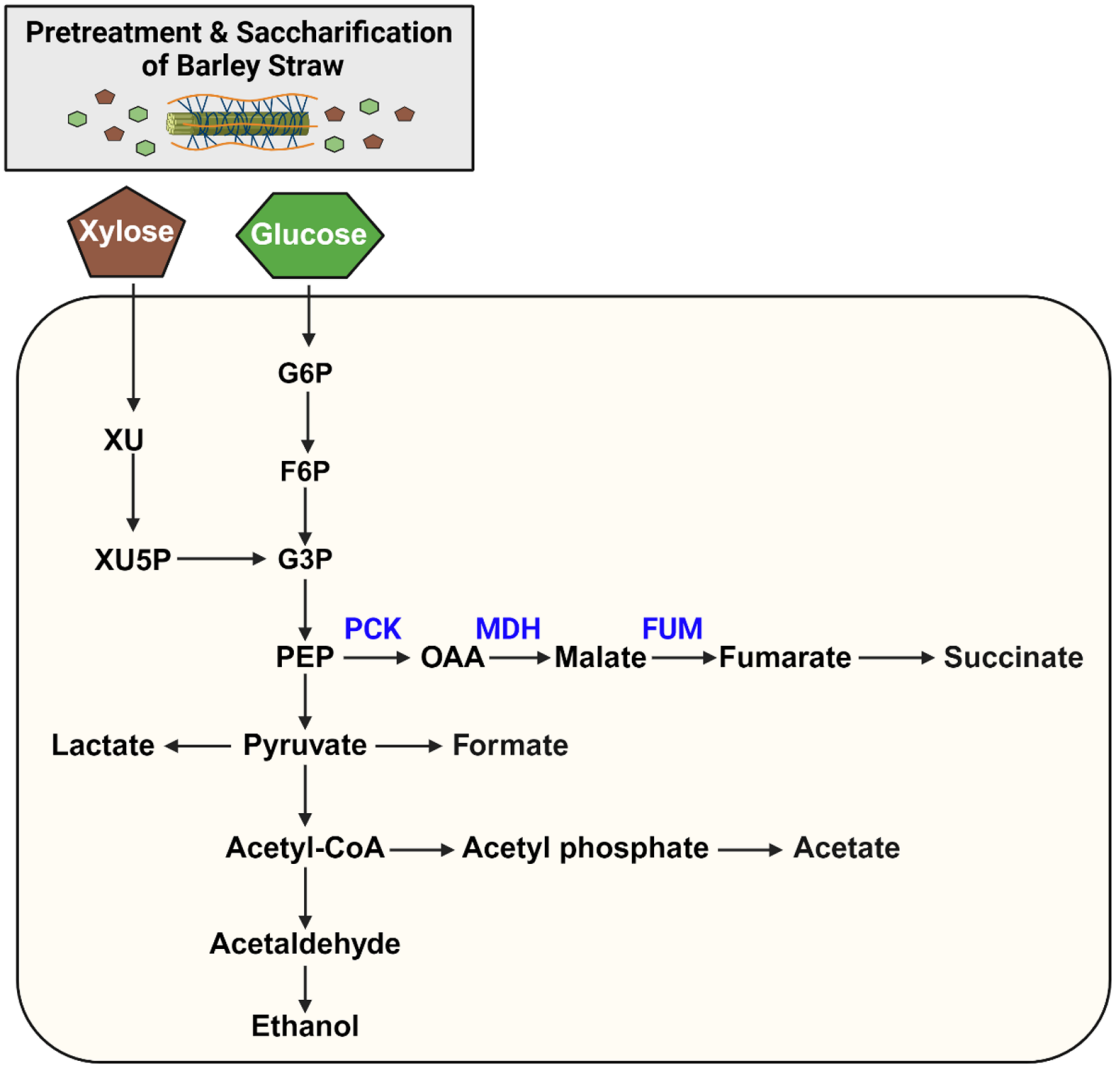
Metabolic pathway of succinate production from hydrolysate of barley straw by engineered *A. succinogenes*. Black arrows carbon flux of xylose and glucose derived from hydrolysate of barley straw. Overexpressed genes to improve succinate production are presented in blue. XU, Xylulose; XY5P, xylulose 5-phosphate; G6P, Glucose 6-phosphate; F6P, Fructose 6-phosphate; G3P, Glyceraldehyde 3-phosphate; PEP, Phosphoenolpyruvate; OAA, oxaloacetate; PCK, Phosphoenol carboxykinase; MDH, malate dehydrogenase; FUM, fumarase.

To improve the ability of SA production, engineered *A. succinogenes* were constructed by introducing plasmids containing genes involved in the SA biosynthetic pathway (Table 1). As shown in Fig. S1, each plasmid was constructed with each gene in the SA biosynthetic pathway with corresponding antibiotics. Each plasmid was transferred into *A. succinogenes* by electroporation to overexpress the target genes. Three genes related to SA production were expressed individually and all together to confirm the improvement of SA production. The engineered strains were *A. succinogenes* AS-P, AS-M, and AS-F to express phosphoenolpyruvate carboxykinase (PCK), malate dehydrogenase (MDH), and fumarase (FUM), respectively (Fig. 1 and Table 1).

### Improved SA Production by Engineered *A. succinogenes*

**Fig. 2:**
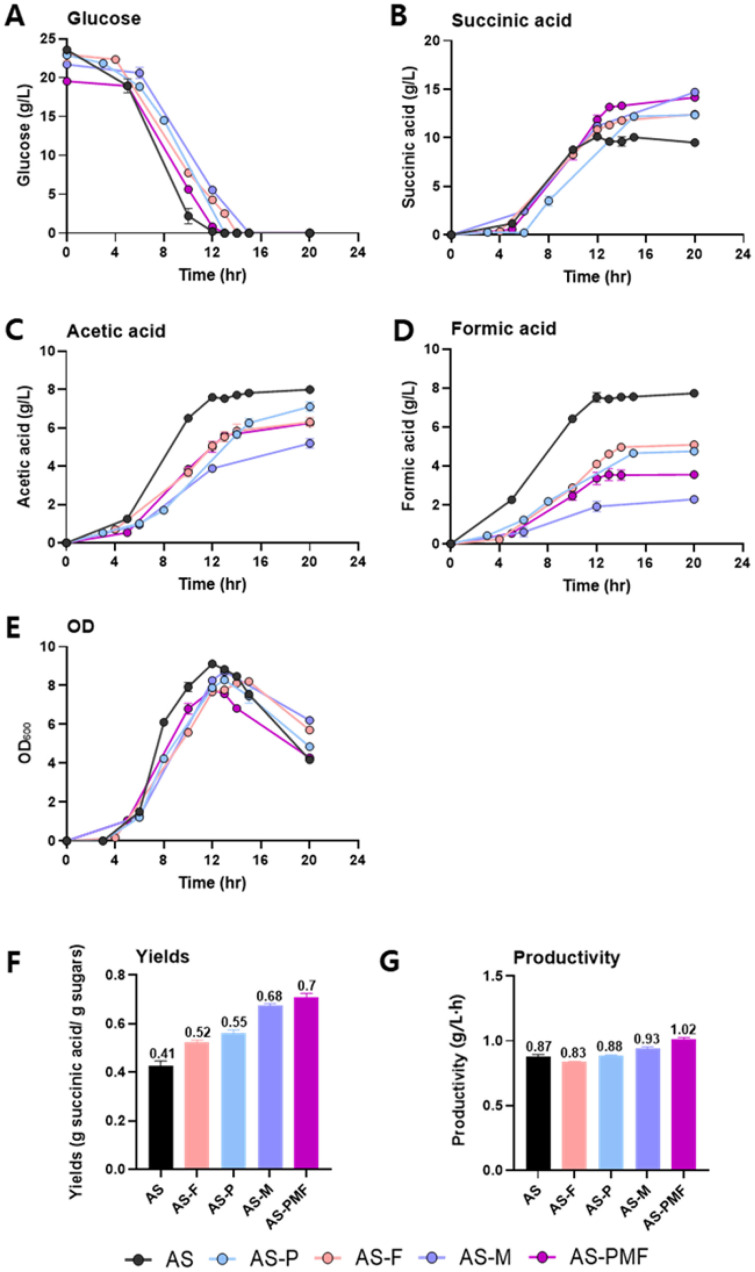
Batch fermentations by engineered *A. succinogenes* overexpressing genes involved in succinate production. Fermentation was performed at 37°C and pH 6.7 in a bioreactor with a working volume of 2.5 L. The pH was maintained at 6.7 by supplying 6 N NaOH. (**A**) Glucose consumption, (**B**) Succinic acid titer, (**C**) Acetic acid titer, (**D**) Formic acid titer, Yield (**E**) and Productivity (**F**) of succinic acid by engineered strains (AS, AS-F, AS-P, AS-M, and AS-PMF).

*A. succinogenes* has two main fermentation pathways based on its carbon flow. One is a reductive C4 pathway that produces SA; the other should be a series of C3 pathways that produce lactate, acetate, formate, and ethanol as end-products (Fig. 1) [31]. Batch fermentation was performed using wild-type and four engineered strains to investigate the effect of overexpression of genes associated with the SA biosynthetic pathway on SA production (Fig. 2). In batch fermentation with glucose as a carbon source, the engineered strains of AS-P, AS-M, and AS-F showed a higher yield of SA (0.55, 0.68, and 0.52 g/g, respectively) compared to their wild-type strain (0.41 g/g)(Fig. 2A and 2F). Especially, a strain AS-M showed the highest final SA titer and yield (Fig. 2B and 2F). It demonstrates that the malate dehydrogenase (MDH) could have an important role in the production of SA. It also proves that oxaloacetate is able to be converted to malate in the reductive TCA cycle of *A. succinogenes* instead of converting malate to oxaloacetate. The AS-PMF strain, where all three genes were expressed, produced ~ 15 g/l of SA and revealed a 1.7-fold increase in yield (0.7 g/g) compared to the wild-type strain when utilizing 20 g/l of glucose (Fig. 2B and 2E). Also, the 1.2-fold higher productivity of SA was achieved in the AS-PMF strain compared with wild-type strain (Fig. 2F and 2G). Furthermore, these engineered strains produced less formic acid and acetic acid than its wild-type strain since carbon flux was towards succinic acid from phosphoenolpyruvate, a starting precursor for SA in those strains overexpressing *pck*, *mdh*, and *fum* genes involved in the SA biosynthetic pathway (Fig. 2C and 2D).

To investigate the ability to produce SA from C5 and C6 sugars as carbon sources, batch fermentations in the medium with 10 g/l xylose and 10 g/l glucose were performed using engineered *A. succinogenes* strains (Table 2). As summarized in Table 2, the AS-PMF, which is a strain overexpressing all three genes, *pck*, *mdh* and *fum*), showed the highest SA yield (0.64 g/g) among tested strains (Table 2). In comparison with batch fermentation with 20 g/l of glucose as a sole carbon source using AS-PMF strain, 20% and 10 % lower SA titer and yield, respectively, was obtained in those with a mixture of 10 g/l xylose and 10 g/l glucose (Fig. 2 and Table 2). These results suggested that *A. succinogenes* preferred to use C6 sugars over C5 sugars.

### Fed-Batch Fermentation with MgCO_3_ as a pH Buffer by an Engineered Strain, *A. succinogenes* AS-PMF

**Fig. 3:**
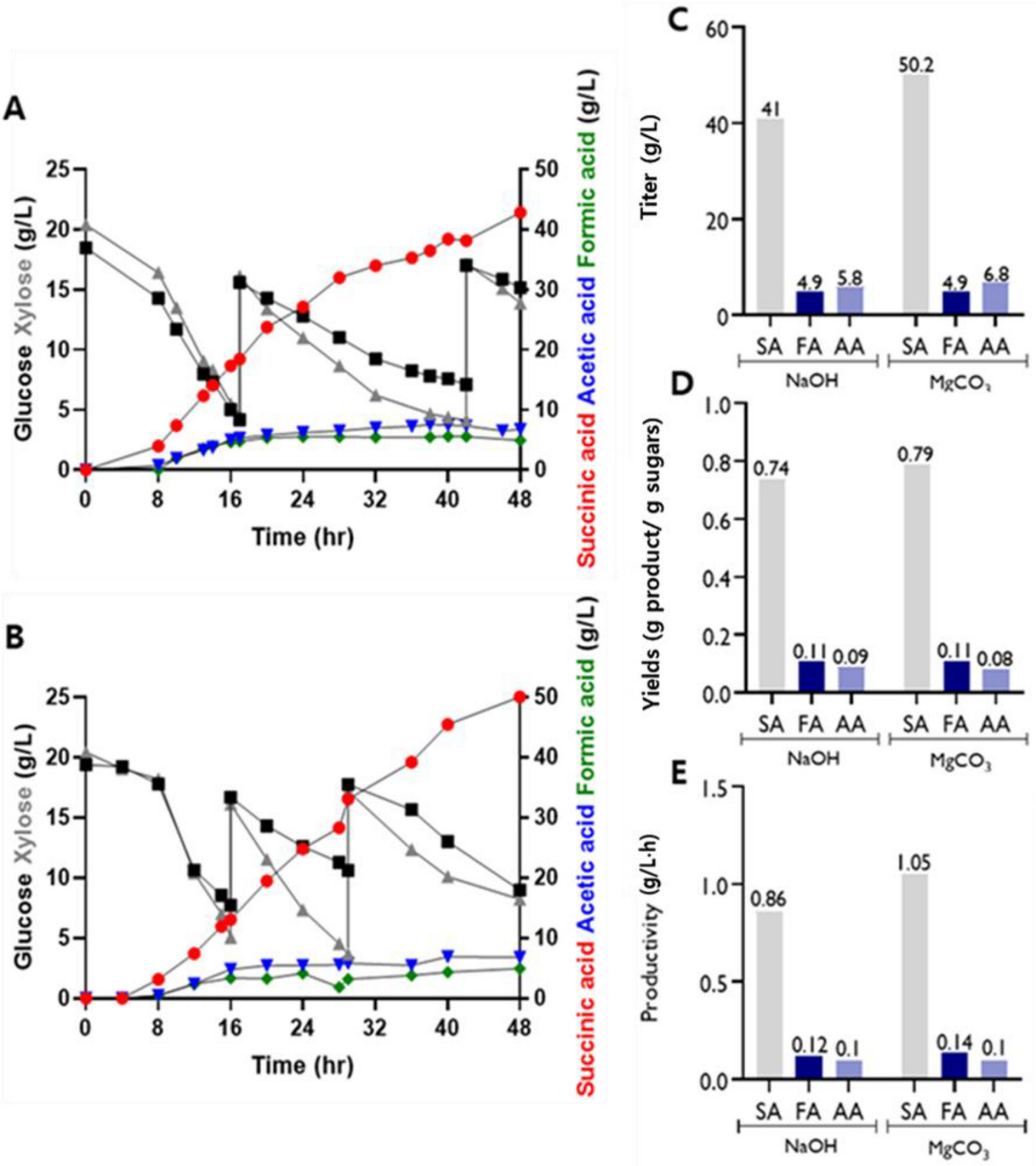
Fed-batch fermentations by the engineered *A. succinogenes* (AS-PMF) with feeding a mixture of glucose and xylose. (**A**) The fermentation profile was maintained during fed-batch fermentation at pH 6.7 with 6N NaOH. (**B**) The fermentation profile was maintained during fed-batch fermentation at pH 6.7 with 50 g/l solid MgCO_3_ as a pH buffer. The bar graphs show the titers of SA, FA, and AA (**C**), yields (**D**), and productivity (**E**) of SA. The productivity of SA was calculated at 48 h. SA: succinic acid, AA: acetic acid, FA: formic acid.

Since the higher SA production was obtained when MgCO_3_ was supplied in the medium as a pH buffer in a previous study [35], the fed-batch fermentation using AS-PMF strain was conducted with 50 g/l solid MgCO_3_ (Fig. 3). The AS-PMF strain produced 41 g/l of SA when 6 N NaOH was supplied in the fed-fermentation by feeding a mixture of glucose and xylose (Fig. 3A), while the strain produced 50 g/l of SA in the fed-batch fermentation with 50 g/l solid MgCO_3_ (Fig. 3B). As shown in Fig. 3C, 3D, and 3E, the strain with MgCO_3_ produced 1.2-fold increased SA with 0.79 g/g SA yield in fed-batch fermentation than that with NaOH as a pH controller.

In usual, NaOH was used as a pH buffer in the acid-producing fermentation because it is a strong base and reacts with free acid to form undissociated water molecules for the neutralization of the solutions. Although NaOH could be a good pH buffer for the fermentation, a previous study reported that NaOH as a pH buffer did not improve SA production [36]. Fermentations using MgCO_3_ as a pH buffer demonstrated that MgCO_3_ could be the most effective pH controlling agent for the growth of *A. succinogenes* because it provides CO_2_ and Mg^2^+ ions that act as co-factors for several enzymes in the SA synthesis pathway [36, 37]. These results demonstrate that MgCO_3_ can contribute to SA production and be an appropriate pH controller.

### SA Production from Lignocellulosic Biomass by an Engineered A. succinogens

Lignocellulosic biomass, especially barley straw, provides carbon sources from its cellulosic materials for the growth of microorganisms. In addition to carbon sources, barley straw also contains and provides other minor components that are not in the cell walls, such as extractable compounds in water and solvents. These minor components from barley straw should be a good source of micronutrients, which are required for the growth of various microorganisms [38, 39]. Other than that, barley straw has another advantage of low collection costs and uniform biomass across varieties because most of barley cultivation area, especially in case of South Korea, is concentrated in one area [40].

Hydrolyzed lignocellulose should be a renewable resource that can be converted to SA and others by fermentation. Specifically, the carbon source from lignocellulosic feedstocks is composed of monosaccharides, such as glucose, xylose, mannose, galactose, and arabinose, which should be non-polluting resources [41, 42]. Because of these environmental and economic reasons as mentioned above, barely straw was used to get the lignocellulosic hydrolysate for the fermentation to produce bio-SA in this study. Pretreatment of lignocellulosic biomass is crucial for saccharification because enzymes cannot directly degrade lignin in plant cell walls. Generally, the barley straw is consisted of ~ 30% hemicellulose, ~ 40% cellulose and ~ 30% lignin [43].

**Fig. 4:**
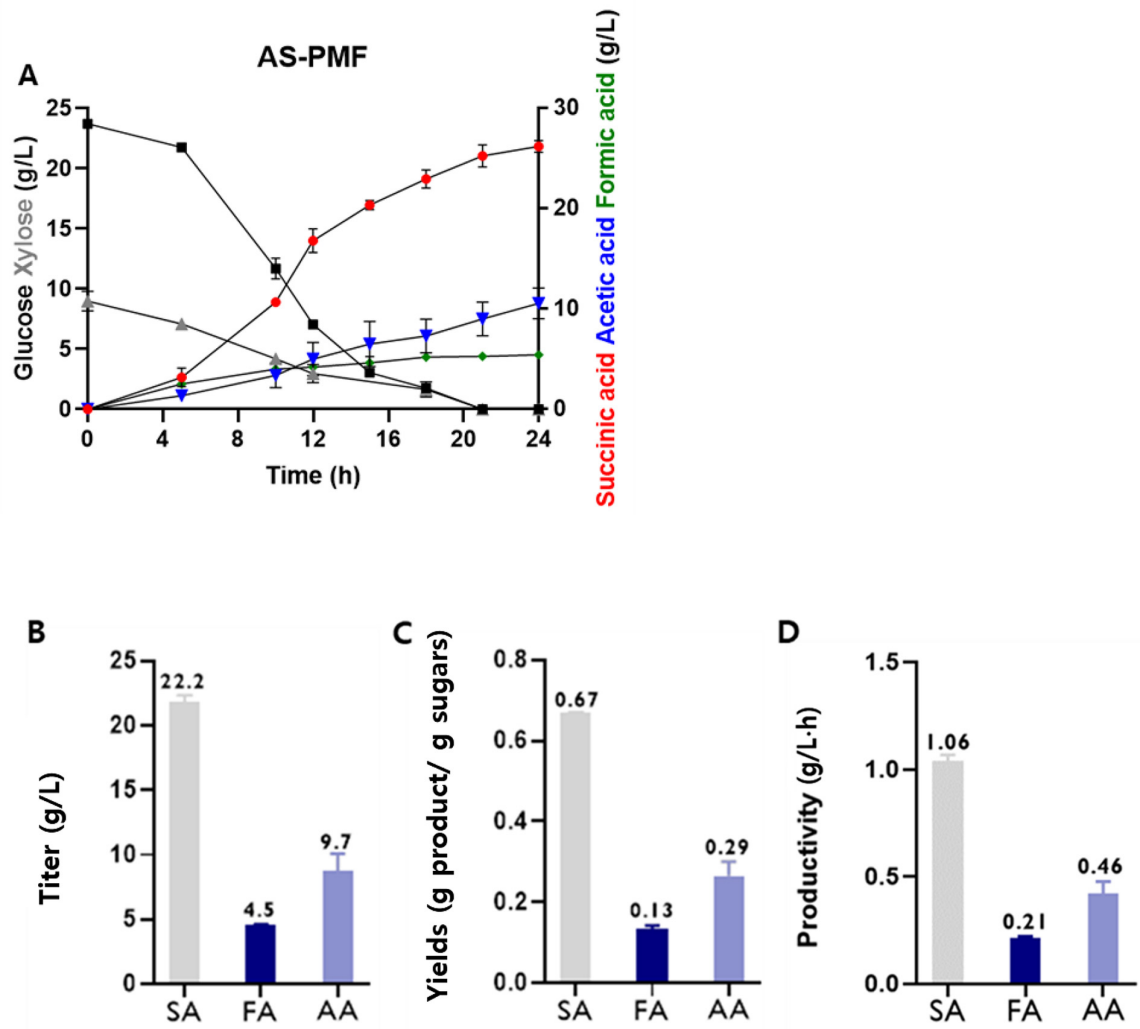
Succinate production by the engineered strain (AS-PMF) using barley straw of lignocellulosic biomass. (**A**) The fermentation profile during batch fermentation. The pH was maintained at 6.7 with 3 N Na_2_CO_3_ and 50 g/l solid MgCO_3_ for buffering pH. Fermentation was carried out at 37°C and pH 6.7, in a bioreactor with 2 L of fermentation medium (23.7 g/l glucose and 9.6 g/l xylose, 13 g/l yeast extract, 19 g/l corn steep liquor, and 19 g/l NaHCO3). The bar graphs show the titers of SA, FA, AA (**B**), yields (**C**) and productivity (**D**) of SA. The productivity was calculated at 21 h. SA: succinic acid, FA: formic acid, AA: acetic acid.

In batch fermentation, the engineered strain, AS-PMF, utilized the lignocellulosic hydrolysate derived from pretreated barley straw as a carbon source. The fermentation medium consisted of 23.7 g/l glucose and 9.6 g/l xylose, derived from the hydrolysate (Fig. 4A). While the carbon source of the medium in the fermentation was come from lignocellulosic hydrolysate, the nitrogen source was provided by 19 g/l corn steep liquor and 13 g/l yeast extract. The *A. succinogenes* AS-PMF produced 22.2 g/l of SA from the lignocellulosic hydrolysate showing a yield of 0.67 g/g and a productivity of 1.16 g/l·h (Fig. 4B, 4C, and 4D). It emonstrates that the engineered strain, AS-PMF, can produce SA from plant wastes although the plants need to be pretreated before the fermentation.

## Conclusion

In this work, a fermentation process was conducted particularly with lignocellulosic biomass, barely straw, as a sole carbon source, and this process was performed by an engineered strain AS-PMF overexpressing *pck*, *mdh*, and *fum* genes involved in the SA biosynthetic pathway. As a result, it showed that this fermentation process produced a relatively high level of SA with a titer of 22.2 g/l and a yield of 0.67 g/g by *A. succinogenes* AS-PMF from the pretreated barley straw. Based on the results in this work, the optimized fermentation condition, specifically using MgCO_3_, both in a batch fermentation and in a fed-batch fermentation, will be useful for the effective production of SA and other industrially important chemicals from various and relatively cheap plant biomass. Furthermore, the production of SA and other useful bio-chemicals can be improved through the overexpression of other genes, which can be genes involved in transport systems. Alternatively, the promoters of genes to be overexpressed can be switched to strong promoters to amplify SA production.

## Supplemental Materials

Supplementary data for this paper are available on-line only at http://jmb.or.kr.



## Figures and Tables

**Table 1:** Strains and plasmids used in this study.

Name	Description	References
Strain		
AS	*A. succinogenes* 130Z (ATCC 55618), Wild type	*ATCC
AS-P	AS derivative harboring pPCK	This study
AS-M	AS derivative harboring pMDH	This study
AS-F	AS derivative harboring pFUM	This study
AS-PMF	AS derivative harboring pPMF	This study
Plasmid		
pPCK	pLGZ920 derivative; Amp^r^; *pckA* gene under the control of *pckA* promoter	Add gene
pMDH	pLGZ920 derivative; Amp^r^; *mdh* gene under the control of promoter *mdh*	Add gene
pPCK-Cm	pLGZ920 derivative; Cm^r^; *pckA* gene under the control of promoter *pckA*	This study
pFUM	pPCK derivative; Amp^r^; *fum* gene under the control of promoter *pckA*	This study
pPMF	pPCK derivative; Cm^r^; *pckA* gene under the control of promoter *pckA*; *mdh* gene under the control of promoter *mdh*; *fum* gene under the control of promoter *fum*	This study

*ATCC: American Type Culture Collection

**Table 2:** Batch fermentation on 10 g/l xylose and 10 g/l by *A. succinogenes* mutant strains (18 h).

Strain	Consumed carbon (g/l)		Concentration (g/l)			Yield (g/g)
	Glucose	Xylose	Succinic acid	Acetic acid	Formic acid	Succinic acid
AS (wild-type)	9.8 ± 0.8	12.0 ± 0.6	10.7 ± 0.1	7.0 ± 0.1	6.3 ± 0.2	0.49 ± 0.05
AS-M	10.9 ± 0.7	12.5 ± 0.9	12.2 ± 0.1	6.6 ± 0.1	5.8 ± 0.1	0.52 ± 0.06
AS-F	10.3 ± 0.8	11.9 ± 1.0	11.3 ± 0.2	7.0 ± 0.1	6.6 ± 0.2	0.51 ± 0.05
AS-PMF	7.9 ± 0.9	12.4 ± 1.2	13.0 ± 0.1	6.8 ± 0.2	5.5 ± 0.1	0.64 ± 0.05

## References

[1] Cha M, Chung D, Westpheling J (2016). Deletion of a gene cluster for [Ni-Fe] hydrogenase maturation in the anaerobic hyperthermophilic bacterium *Caldicellulosiruptor bescii* identifies its role in hydrogen metabolism. Appl. Microbiol. Biotechnol..

[2] Cha M, Kim JH, Choi HJ, Nho SB, Kim SY, Cha YL (2023). Hydrogen production from barley straw and miscanthus by the hyperthermophilic bacterium, *Cadicellulosirupter bescii*. J. Microbiol. Biotechnol..

[3] Chung D, Cha M, Guss AM, Westpheling J (2014). Direct conversion of plant biomass to ethanol by engineered *Caldicellulosiruptor bescii*. Proc. Natl. Acad .Sci. USA.

[4] Werpy T, Petersen G Top Value Added Chemicals From Biomass 2004, Pacific Northwest National Laboratory (PNNL).

[5] Goldberg I, Rokem JS, Pines O (2006). Organic acids: old metabolites, new themes. J. Chem. Technol. Biotechnol..

[6] Jampatesh S, Sawisit A, Wong N, Jantama SS, Jantama K (2019). Evaluation of inhibitory effect and feasible utilization of dilute acidpretreated rice straws on succinate production by metabolically engineered *Escherichia coli* AS1600a. Bioresour. Technol..

[7] Xiao N, Lou MD, Lu YT, Yang LL, Liu Q, Liu B (2017). Ginsenoside Rg5 attenuates hepatic glucagon response via suppression of succinate-associated HIF-1alpha induction in HFD-fed mice. Diabetologia.

[8] Aliotta L, Seggiani M, Lazzeri A, Gigante V, Cinelli P (2022). A brief review of poly (Butylene Succinate) (PBS) and its main copolymers: synthesis, blends, composites, biodegradability, and applications. Polymers (Basel).

[9] Armani D, Petri A (2023). Enzymes in poly(Butylene-Succinate) industry: an overview on synthesis routes and post-processing strategies. Catal. Res..

[10] Abd Alsaheb RA, Atyia MA, Abdullah JK, Abbas AH (2023). Succinic acid production strategy: raw material, organisms and recent applications in pharmaceutical and food: critical review. Al-Khwarizmi Eng. J..

[11] Salma A, Djelal H, Abdallah R, Fourcade F, Amrane A (2021). Platform molecule from sustainable raw materials; case. study succinic acid. Braz. J. Chem. Eng..

[12] Cok B, Tsiropoulos I, Roes AL, Patel MK (2013). Succinic acid production derived from carbohydrates: an energy and greenhouse gas assessment of a platform chemical toward a bio-based economy. Biofuels Bioprod. Biorefin..

[13] Mitrea L, Teleky BE, Nemes SA, Plamada D, Varvara RA, Pascuta MS (2024). Succinic acid - A run-through of the latest perspectives of production from renewable biomass. Heliyon.

[14] Zeikus JG, Jain MK, Elankovan P (1999). Biotechnology of succinic acid production and markets for derived industrial products. Appl. Microbiol. Biotechnol..

[15] Beauprez JJ, Mey MD, Soetaert WK (2010). Microbial succinic acid production: natural versus metabolic engineered producers. Process Biochem..

[16] Ahn JH, Jang YS, Lee SY (2016). Production of succinic acid by metabolically engineered microorganisms. Curr. Opin. Biotechnol..

[17] Liu X, Zhao G, Sun S, Fan C, Feng X, Xiong P (2022). Biosynthetic pathway and metabolic engineering of succinic acid. Front. Bioeng. Biotechnol..

[18] Isar J, Agarwal L, Saran S, Kaushik R, Saxena RK (2007). A statistical approach to study the interactive effects of process parameters on succinic acid production from Bacteriodes fragilis. Anaerobe.

[19] Okino S, Noburyu R, Suda M, Jojima T, Inui M, Yukawa H (2008). An efficient succinic acid production process in a metabolically engineered *Corynebacterium glutamicum* strain. Appl. Microbiol. Biotechnol..

[20] Stephanopoulos G, Aristidou AA, Nielsen J (1998). Metabolic engineering. Principles and methodologies. 1998.

[21] Wang D, Li Q, Song Z, Zhou W, Su Z, Xing J (2010). High cell density fermentation via a metabolically engineered *Escherichia coli* for the enhanced production of succinic acid. J. Chem. Technol. Biotechnol..

[22] Sanchez AM, Bennett GN, San KY (2005). Novel pathway engineering design of the anaerobic central metabolic pathway in *Escherichia coli* to increase succinate yield and productivity. Metab. Eng..

[23] Lin H, Bennett GN, San KY (2005). Metabolic engineering of aerobic succinate production systems in *Escherichia coli* to improve process productivity and achieve the maximum theoretical succinate yield. Metab. Eng..

[24] Liu R, Liang L, Cao W, Wu M, Chen K, Ma J, Jiang M (2013). Succinate production by metabolically engineered *Escherichia coli* using sugarcane bagasse hydrolysate as the carbon source. Bioresour. Technol..

[25] Ahn JH, Seo H, Park W, Seok J, Lee JA, Kim WJ (2020). Enhanced succinic acid production by Mannheimia employing optimal malate dehydrogenase. Nat. Commun..

[26] Van der Werf MJ, Guettler MV, Jain MK, Zeikus JG (1997). Environmental and physiological factors affecting the succinate product ratio during carbohydrate fermentation by *Actinobacillus* sp. 130Z. Arch. Microbiol..

[27] Guettler MV, Rumler D, Jain MK (1999). *Actinobacillus succinogenes* sp. nov., a novel succinic-acid-producing strain from the bovine rumen. Int. J. Syst. Bacteriol. 49 Pt.

[28] Du C, Lin SKC, Koutinas A, Wang R, Dorado P, Webb C (2008). A wheat biorefining strategy based on solid-state fermentation for fermentative production of succinic acid. Bioresour. Technol..

[29] Liu YP, Zheng P, Sun ZH, Ni Y, Dong JJ, Zhu LL (2008). Economical succinic acid production from cane molasses by *Actinobacillus succinogenes*. Bioresour. Technol..

[30] Wan C, Li Y, Shahbazi A, Xiu S (2008). Succinic acid production from cheese whey using *Actinobacillus succinogenes* 130 Z. Appl. Biochem. Biotechnol..

[31] Guarnieri MT, Chou YC, Salvachúa D, Mohagheghi A, St John PC, Peterson DJ (2017). Metabolic engineering of *Actinobacillus succinogenes* provides insights into succinic acid biosynthesis. Appl. Environ. Microbiol..

[32] McKinlay JB, Laivenieks M, Schindler BD, McKinlay AA, Siddaramappa S, Challacombe JF (2010). A genomic perspective on the potential of *Actinobacillus succinogenes* for industrial succinate production. BMC Genomics.

[33] Dessie W, Zhang W, Xin F, Dong W, Zhang M, Ma J (2018). Succinic acid production from fruit and vegetable wastes hydrolyzed by on-site enzyme mixtures through solid state fermentation. Bioresour. Technol..

[34] Joshi RV, Schindler BD, McPherson NR, Tiwari K, Vieille C (2014). Development of a markerless knockout method for *Actinobacillus succinogenes*. Appl. Environ. Microbiol..

[35] Yu J, Li Z, Ye Q, Yang Y, Chen S (2010). Development of succinic acid production from corncob hydrolysate by *Actinobacillus succinogenes*. J. Ind. Microbiol. Biotechnol..

[36] Dessie W, Xin F, Zhang W, Jiang Y, Wu H, Ma J (2018). Opportunities, challenges, and future perspectives of succinic acid production by *Actinobacillus succinogenes*. Appl. Microbiol. Biotechnol..

[37] Wang CC, Zhu LW, Li HM, Tang YJ (2012). Performance analyses of a neutralizing agent combination strategy for the production of succinic acid by *Actinobacillus succinogenes* ATCC 55618. Bioprocess Biosyst. Eng..

[38] Toledano A, García A, Mondragon I, Labidi J (2010). Lignin separation and fractionation by ultrafiltration. Sep. Purif. Technol..

[39] Serna-Díaz MG, Mercado-Flores Y, Jiménez-González A, Anducho-Reyes MA, Medina-Marín J, Tuoh-Mora JCS (2010). Use of barley straw as a support for the production of conidiospores of *Trichoderma harzianum*. Biotechnol. Rep..

[40] Kim Y, Yu A, Chung B, Han M, Choi G (2009). Lignin removal from barley straw by ethanosolv pretreatment. Korean Soc. Biotechnol. Bioeng J..

[41] Poletto P, Pereira GN, Monteiro CRM, Pereira MAF, Bordignon SE, de Oliveira D (2020). Xylooligosaccharides production process from lignocellulosic biomass and bioactive effects. Process Biochem..

[42] Isikgor FH, Becer CR (2015). Lignocellulosic biomass: a sustainable platform for the production of bio-based chemicals and polymers. Polym. Chem..

[43] Gan I, Chow WS (2018). Antimicrobial poly(lactic acid)/cellulose bionanocomposite for food packaging application: a review. Food Packaging Shelf Life.

