# Supplementary material for: Production of Succinic Acid by Metabolically Engineered Actinobacillus succinogenes from Lignocellulosic Hydrolysate Derived from Barley Straw
Source: J Microbiol Biotechnol. 2024 Nov 25;34(12):2618–26. doi: 10.4014/jmb.2410.10053 (PMC11729488; doi:10.4014/jmb.2410.10053)
Supplement: Supplementary file 1 [file jmb-34-12-2618-supple.pdf]

J. Microbiol. Biotechnol. 2024. 34(12): 2618–2626
https://doi.org/10.4014/jmb.2410.10053

Supplementary Figure and Tables

Production of succinic acid by metabolically engineered *Actinobacillus succinogenes* strains from lignocellulosic hydrolysate derived from barley straw

Bo-Kyung Kim[1], Min-Seo Park[1], Minseok Cha[2], Young-Lok Cha[3], and Soo-Jung Kim[1,2]*

[1] Department of Integrative Food, Bioscience and Biotechnology, Chonnam National University, Gwangju 61186, Republic of Korea

[2] Research Center for Biological Cybernetics, Chonnam National University, Gwangju 61186, Republic of Korea

[3] Bioenergy Crop Research Institute, National Institute of Crop Science, Rural Development Administration, Muan 58545, Republic of Korea

* Correspondence to:

Soo-Jung Kim

Email: bioksj@jnu.ac.kr

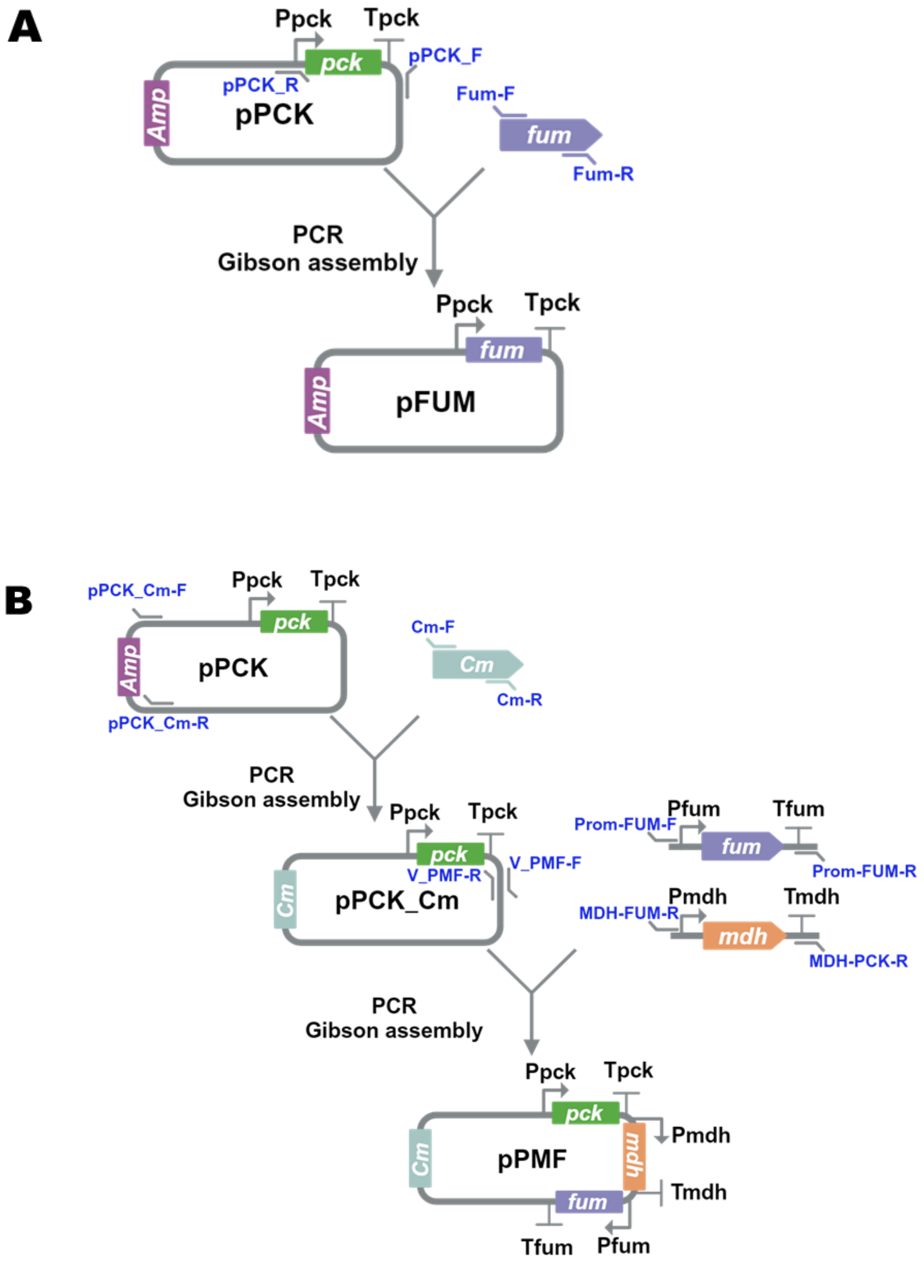

Fig. S1. Construction of plasmids used in this study. (A) pFUM; **(B)** pPMF. Primers listed in Table S1 are presented in blue.

Table S1. Primers to construct plasmids in this study.

Constructed plasmid	Fragment name	Template	Primer	Sequence
pFUM	V_PCK	PCK plasmid	pPCK-F	TGGTTGGCAGCTTAAAATAAAACTGTAAAAGCA TAGTATGTGC
			pPCK-R	ACTTCACCTCATTGATAATTTAA
	I_FUM	*A. succinogenes* genomic DNA	FUM-F	TTATTTTAAGCTGCCAACCA
			FUM-R	AATTATCAATGAGGTGAAGTATGACATTTCGTA TTGAAAA
pPCK_Cm	V_PCK_Cm	PCK plasmid	pPCK_Cm-F	CTGTCAGACCAAGTTTACTC
			pPCK_Cm-R	ACTCTTCCTTTTTCAATATT
	I_Cm	*cm* synthetic gene	Cm-F	AATATTGAAAAAGGAAGAGTATGGAGAAAAAAA TCACTGG
			Cm-R	GAGTAAACTTGGTCTGACAGTTACGCCCCGCC CTGCCACT
pPMF	V_PMF	pPCK_Cm	V_PMF-F	GCGAGGAAAAGCAAACTGTAAAAGCATAGTAT GTGCAT
			V_PMF-R	GGCGAATAATTATGCTTTTGGACCGGCG
	I_Promoter_MDH	*A. succinogenes* genomic DNA	MDH-FUM-R	CTTCGGCAAACCGGTGGAAAAATTCTGCATAAA AATTCAA
			MDH-PCK-R	GTCCAAAAGCATAATTATTCGCCGGTAACAAAT TTCACA
	I_Promoter_FUM	*A. succinogenes* genomic DNA	Prom-FUM-F	AGAATTTTTCCACCGGTTTGCCGAAGT
			Prom-FUM-R	CTTTTACAGTTTGCTTTTCCTCGCGTTCATC

Table S2. Primers to confirm constructed plasmids in this study.

Plasmid	Primer	Sequence
pFUM	pPCK-col-F	GAGTGAGCTGATACCGCTCG
	pPCK_col-R	TCCAAAACAAAAGGCTGAGTGG
pPCK-Cm	pPCK_Cm_col-F	AATATTGAAAAAGGAAGAGTATGGAGAAAAAAATCACTGG
	pPCK_Cm_col-R	GAGTAAACTTGGTCTGACAGTTACGCCCCGCCCTGCCACT
pPMF	PMF_Cm_col_1_F	GTGAAAAACACCGTATCGGTGT
	PMF_Cm_col_2_F	GCGGACGCATTCGGCGTATTG
	PMF_Cm_col_3_F	TACTACGCTGGATACCATCCGT
	PMF_Cm_col_4_F	GATACCAGAACGAGGACCTGA

Table S3. Gene sequences used in plasmid constructions.

Gene	Sequence
fum	ATGACATTTCGTATTGAAAAAGACACTATGGGCGAGGTTCAAGTTCCTGCGGATAAATATTGGGCCGCACAAACGGAACGTTCACGC<br>AATAACTTTAAAATCGGACCTGCAGCGTCAATGCCACACGAAATTATTGAAGCATTCGGTTATTTGAAAAAAGCGGCCGCTTATGCCA<br>ACGCGGATTTAGGCGTATTGCCGGCTGAAAAACGCGATTTGATCGCACAAGCCTGCGATGAAATTCTGGCTCGTAAATTAGATGATC<br>AATTCCCGTTAGTTATCTGGCAAACCGGTTCGGGTACACAATCCAATATGAATCTGAACGAAGTTATCGCTAACCGCGCACATGTGAT<br>TAACGGTGGTAAATTAGGTGAAAAATCTATTATTCATCCAAACGACGATGTAAACAAATCTCAATCTTCAAACGATACTTATCCGACAG<br>CAATGCACATTGCCACATATAAGAAAGTGGTTGAAGCAACGATTCCGGCCATCGAACGTTTACAAAAAACCTTAGCGGCGAAATCCG<br>AAGAATTCAAAGATGTGGTGAAAATCGGCCGTACGCACTTAATGGATGCCACCCCGTTGACATTGGGTCAGGAATTCAGCGGTTATG<br>CTGCACAATTAAGTTTCGGTTTAGCGGCAATCAAAAATACCTTACCGCATTTACGCCAACTGGCATTAGGCGGTACGGCAGTGGGTA<br>CCGGTTTAAATACACCTAAAGGCTATGATGTAAAAGTAGCGGAATATATCGCCAAATTCACCGGCTTGCCGTTTATTACCGCCGAAAA<br>CAAATTTGAAGCATTAGCAACACATGACGCTATCGTTGAAACTCACGGCGCATTAAAACAAGTTGCGATGTCCTTATTCAAAATTGCAA<br>ATGATATCCGTTTATTGGCTTCAGGTCCTCGTTCTGGTATCGGTGAAATTTTAATTCCTGAAAACGAACCGGGTTCATCCATCATGCC<br>GGGTAAAGTTAATCCGACCCAATGCGAAGCGATGACAATGGTTGCCGCACAAGTATTAGGTAACGATACCACTATTTCATTTGCCGGT<br>TCGCAAGGTCATTTCGAATTGAACGTATTCAAACCGGTTATGGCGGCAAATTTCCTGCAATCCGCTCAATTAATCGCAGATGTTTGCA<br>TTTCTTTCGACGAGCACTGTGCAAGCGGCATTAAACCAAATACGCCGCGCATTCAACACTTACTTGAAAGTTCATTAATGTTAGTGAC<br>CGCATTAAATACTCATATCGGTTATGAAAATGCGGCGAAAATTGCGAAAACTGCGCACAAAAACGGTACAACATTACGTGAAGAGGCT<br>ATCAACTTAGGTTTAGTGTCCGCCGAAGATTTCGATAAATGGGTTCGTCCGGAAGATATGGTTGGCAGCTTAAAATAA
pckA	ATGACTGACTTAAACAAACTCGTTAAAGAACTTAATGACTTAGGGCTTACCGATGTTAAGGAAATTGTGTATAACCCGAGTTATGAACA<br>ACTTTTCGAGGAAGAAACCAAACCGGGTTTGGAGGGTTTCGATAAAGGGACGTTAACCACGCTTGGCGCGGTTGCCGTCGATACGG<br>GGATTTTTACCGGTCGTTCACCGAAAGATAAATATATCGTTTGCGATGAAACTACGAAAGACACCGTTTGGTGGAACAGCGAAGCGG<br>CGAAAAACGATAACAAACCGATGACGCAAGAAACTTGGAAAAGTTTGAGAGAATTAGTGGCGAAACAACTTTCCGGTAAACGTTTATT<br>CGTGGTAGAAGGTTACTGCGGCGCCAGTGAAAAACACCGTATCGGTGTGCGTATGGTTACTGAAGTGGCATGGCAGGCGCATTTTG<br>TGAAAAACATGTTTATCCGACCGACCGATGAAGAGTTGAAAAATTTCAAAGCGGATTTTACCGTGTTAAACGGTGCTAAATGTACTAAT<br>CCGAACTGGAAAGAACAAGGTTTGAACAGTGAAAACTTTGTCGCTTTCAATATTACCGAAGGTATTCAGCTTATCGGCGGTACTTGGT<br>ACGGCGGTGAAATGAAAAAAGGTATGTTCTCAATGATGAACTACTTCCTGCCGTTAAAAGGTGTGGCTTCCATGCACTGTTCCGCCAA<br>CGTAGGTAAAGACGGTGACGTGGCTATTTTCTTCGGTTTATCCGGTACGGGTAAAACAACGCTTTCGACCGATCCTAAACGCCAATTA<br>ATCGGTGATGACGAACACGGTTGGGATGAATCCGGCGTATTTAACTTTGAAGGCGGTTGTTACGCGAAAACCATTAACTTATCTCAAG

	AAAACGAACCGGATATTTACGGCGCAATCCGTCGTGACGCATTATTAGAAAACGTCGTGGTTCGTGCAGACGGTTCCGTTGACTTTG<br>ACGACGGTTCAAAAACAGAAAATACCCGTGTTTCATATCCGATTTACCACATCGACAACATCGTTCGTCCGGTATCGAAAGCCGGTCA<br>TGCAACCAAAGTGATTTTCTTAACCGCGGACGCATTCGGCGTATTGCCGCCGGTTTCAAAACTGACTCCGGAACAAACCGAATACTA<br>CTTCTTATCCGGCTTTACTGCAAAATTAGCGGGTACGGAACGCGGCGTAACCGAACCGACTCCGACATTCTCGGCCTGTTTCGGTGC<br>GGCATTCTTAAGCCTGCATCCGATTCAATATGCGGACGTGTTGGTCGAACGCATGAAAGCCTCCGGTGCGGAAGCTTATTTGGTGAA<br>CACCGGTTGGAACGGCACGGGTAAACGTATTTCAATCAAAGATACCCGCGGTATTATCGATGCGATTTTGGACGGTTCAATCGAAAA<br>AGCGGAAATGGGCGAATTGCCAATCTTTAATTTAGCGATTCCTAAAGCATTACCGGGTGTTGATCCTGCTATTTTGGATCCGCGCGAT<br>ACTTACGCAGACAAAGCGCAATGGCAAGTTAAAGCGGAAGATTTGGCAAACCGTTTCGTGAAAAACTTTGTGAAATATACGGCGAATC<br>CGGAAGCGGCTAAATTAGTTGGCGCCGGTCCAAAAGCATAA
mdh	ATGAAAGTAACCTTATTAGGCGCCAGCGGCGGTATCGGTCAACCTCTTTCATTGTTGTTAAAATTACATCTTCCGGCAGAAAGCGATT<br>TAAGCTTATACGATGTTGCGCCGGTCACCCCCGGTGTGGCGAAAGACATCAGCCATATTCCGACTTCGGTTGAAGTGGAAGGTTTCG<br>GCGGCGATGATCCGTCCGAGGCATTAAAAGGGGCGGATATCGTTTTAATCTGTGCGGGTGTGGCGCGTAAGCCGGGTATGACTCGT<br>GCGGATTTGTTTAATGTTAACGCCGGTATTATCCAGAATTTAGTGGAAAAAGTTGCGCAAGTTTGCCCGCAGGCTTGTGTTTGCATTA<br>TCACTAATCCGGTGAACTCGATTATTCCGATTGCGGCGGAAGTGCTGAAAAAAGCGGGCGTATACGATAAACGGAAATTATTCGGTA<br>TTACTACGCTGGATACCATCCGTTCCGAAAAATTTATCGTGCAAGCGAAAAATATTGAAATCAACCGTAACGATATTTCAGTTATCGGC<br>GGACATTCAGGTGTGACGATTTTACCTTTGTTGTCACAAATTCCGCATGTGGAATTTACCGAGCAGGAATTAAAAGATTTAACTCACC<br>GCATCCAAAATGCCGGCACCGAAGTGGTAGAAGCTAAAGCCGGTGCGGGTTCCGCTACACTTTCCATGGCGTATGCGGCAATGCGT<br>TTTGTGGTTTCCATGGCTCGCGCATTAAACGGCGAAGTGATTACGGAATGCGCCTATATTGAAGGCGACGGTAAATTCGCCCGTTTC<br>TTTGCACAACCGGTTCGTTTGGGTAAAAACGGCGTAGAAGAAATTCTGCCGTTAGGTACATTAAGCGCATTTGAGCAACAAGCGCTT<br>GAAGCGATGTTACCGACCTTGCAAACTGACATTGATAACGGTGTGAAATTTGTTACCGGCGAATAA
Cm	ATGGAGAAAAAAATCACTGGATATACCACCGTTGATATATCCCAATGGCATCGTAAAGAACATTTTGAGGCATTTCAGTCAGTTGCTCA<br>ATGTACCTATAACCAGACCGTTCAGCTGGATATTACGGCCTTTTTAAAGACCGTAAAGAAAAATAAGCACAAGTTTTATCCGGCCTTTA<br>TTCACATTCTTGCCCGCCTGATGAATGCTCATCCGGAATTCCGTATGGCAATGAAAGACGGTGAGCTGGTGATATGGGATAGTGTTC<br>ACCCTTGTTACACCGTTTTCCATGAGCAAACTGAAACGTTTTCATCGCTCTGGAGTGAATACCACGACGATTTCCGGCAGTTTCTACA<br>CATATATTCGCAAGATGTGGCGTGTTACGGTGAAAACCTGGCCTATTTCCCTAAAGGGTTTATTGAGAATATGTTTTTCGTCTCAGCCA<br>ATCCCTGGGTGAGTTTCACCAGTTTTGATTTAAACGTGGCCAATATGGACAACTTCTTCGCCCCCGTTTTCACCATGGGCAAATATTAT<br>ACGCAAGGCGACAAGGTGCTGATGCCGCTGGCGATTCAGGTTCATCATGCCGTTTGTGATGGCTTCCATGTCGGCAGAATGCTTAAT<br>GAATTACAACAGTACTGCGATGAGTGGCAGGGCGGGGCGTAA
Amp	ATGAGTATTCAACATTTCCGTGTCGCCCTTATTCCCTTTTTTGCGGCATTTTGCCTTCCTGTTTTTGCTCACCCAGAAACGCTGGTGAA<br>AGTAAAAGATGCTGAAGATCAGTTGGGTGCACGAGTGGGTTACATCGAACTGGATCTCAACAGCGGTAAGATCCTTGAGAGTTTTCG

CCCCGAAGAACGTTTTCCAATGATGAGCACTTTTAAAGTTCTGCTATGTGGCGCGGTATTATCCCGTGTTGACGCCGGGCAAGAGCA
ACTCGGTCGCCGCATACACTATTCTCAGAATGACTTGGTTGAGTACTCACCAGTCACAGAAAAGCATCTTACGGATGGCATGACAGTA
AGAGAATTATGCAGTGCTGCCATAACCATGAGTGATAACACTGCGGCCAACTTACTTCTGACAACGATCGGAGGACCGAAGGAGCTA
ACCGCTTTTTTGCACAACATGGGGGATCATGTAACTCGCCTTGATCGTTGGGAACCGGAGCTGAATGAAGCCATACCAAACGACGAG
CGTGACACCACGATGCCTGCAGCAATGGCAACAACGTTGCGCAAACTATTAACTGGCGAACTACTTACTCTAGCTTCCCGGCAACAA
TTAATAGACTGGATGGAGGCGGATAAAGTTGCAGGACCACTTCTGCGCTCGGCCCTTCCGGCTGGCTGGTTTATTGCTGATAAATCT
GGAGCCGGTGAGCGTGGATCTCGCGGTATCATTGCAGCACTGGGGCCAGATGGTAAGCCCTCCCGTATCGTAGTTATCTACACGAC
GGGGAGTCAGGCAACTATGGATGAACGAAATAGACAGATCGCTGAGATAGGTGCCTCACTGATTAAGCATTGGTAA

Table S4. Promoter sequences used in plasmid constructions.

Gene	Sequence
fum promoter (predicted)	TTTGCTTTTCCTCGCGTTCATCGTCAAGTGCGGTCAAATCCGGGATAATTTCTGCCGTTTCGGA AAACTCACGCTTTACCCGAAAAACCTCTTCTTCATTACCGGCGTTATTTTCTACATGATTGAATA CCCGTTTATCGTTCATTTTTTTCTCCTGTTTTTGTGATTTCAGCAAAAATTACCCGTATACCGATT TAATGATCTAAATCACAAAAACTGTAATTCGTCCTATCCGACCTAACTCCGAAGTGTTAGAATCG ACGTATATTTTACTTTAATCCAATAATAGATAGGTGATTATT
pckA promoter	TCGATAAATTGAAAATGCAGCAATAGAGGAAACACGGTTTGTTTGAGTGAAAACAGCCGTGTTT TTTCATTTACCGCCATAAAAATTTGAAACGGATCACAAATCATGAAAAAAATACGTTCAAATTAG AACTAATTATCGAAAATTTGATCTAGTTAACATTTTTTAGGTATAAATAGTTTTAAAATAGATCTA GTTTGGATTTTTAATTTTAAATTATCAATGAGGTGAAGT
mdh promoter	AAATTCTGCATAAAAATTCAAAATTAATCAATAAAAATTTAAGTTTATTGTGATTTGAGCGTTTTC GAAAAATAAATGATAAAAACTTGTTTTAGATCGTAAAAATAGATGAATATTTAATTGAGTTTCATT TTTTTTCTTCGTAAAATCTACCCAGTTCAAGTTATTAATATTATCGAGGAGTATCTC
Bla promoter	AATTCTTGAAGACGAAAGGGCCTCGTGATACGCCTATTTTTATAGGTTAATGTCATGATAATAAT GGTTTCTTAGACGTCAGGTGGCACTTTTCGGGGAAATGTGCGCGGAACCCCTATTTGTTTATTT TTCTAAATACATTCAAATATGTATCCGCTCATGAGACAATAACCCTGATAAATGCTTCAATAATAT TGAAAAAGGAAGAGT
